# Sampling Ozone Exposure of Canadian Forests at Different Scales: Some Case Studies

**DOI:** 10.1100/tsw.2001.346

**Published:** 2001-12-06

**Authors:** R.M. Cox, J.W. Malcolm, R.N. Hughes, T.P.W. Williams

**Affiliations:** Natural Resources Canada-Canadian Forest Service, Atlantic Forestry Centre, Fredericton, N.B., Canada

**Keywords:** passive ozone sampler, CanOxy Plate, monitoring, air quality, ozone, Acadian mixed forest

## Abstract

The use of passive samplers in extensive monitoring, such as that used in national forest health monitoring plots, indicates that these devices are able to determine both spatial and temporal differences in ozone exposure of the plots. This allows for categorisation of the plots and the potential for cause-effect analysis of certain forest health responses. Forest exposure along a gradient of air pollution deposition demonstrates large variation in accumulated exposures. The efficacy of using passive samplers for in situ monitoring of forest canopy exposure was also demonstrated. The sampler data produced weak relationships with ozone values from the nearest "continuous" monitor, even though data from colocated samplers showed strong relationships. This spatial variation and the apparent effect of elevation on ozone exposure demonstrate the importance of topography and tree canopy characteristics in plant exposure on a regional scale. In addition, passive sampling may identify the effects of local pollutant gases, such as NO, which may scavenge ozone locally only to increase the production of this secondary pollutant downwind, as atmospheric reactions redress the equilibrium between concentrations of this precursor and those of the generated ozone. The use of passive samplers at the stand level is able to resolve vertical profiles within the stand and edge effects that are important in exposure of understorey and ground flora. Recent case studies using passive samplers to determine forest exposure to ozone indicate a great potential for the development of spatial models on a regional, landscape, and stand level scale.

## INTRODUCTION

The air quality objective for ozone, established for the protection of crops and other plants (and other environmental components), is regularly exceeded over large forested areas of Canada, giving rise to the need for both extensive and intensive monitoring programs to determine long-term critical exposure levels for forest damage. Currently, air quality monitoring is being carried out at “continuous” monitoring stations. However, these are limited to a few urban sites and fewer rural sites. The requirement for an inexpensive passive sampler for atmospheric oxidants (specifically ozone) that can be used in remote locations prompted the development of the CanOxy Plate™ passive ozone sampler by the air pollution research group of the Forest Health Network of the Canadian Forest Service. This badge-type sampler consists of an Indigo absorbent covered with a diffusion membrane[1]. Operational application of these samplers in forest plots was implemented from 1997–2001. This involved the simultaneous exposure of these samplers in the upper canopy at selected forest health monitoring plots across the country. More recently, this was also done at forest health plots on a transect across different ozone exposures and climates from central to eastern Canada.

The earliest measurements of ground-level ozone were made by the Swiss chemist Schönbein in the mid-1800s, using test papers impregnated with potassium iodide (cited in London[2]). Other ozone samplers with different reactants have been developed since the 1960s and are shown in Table 1. A few absorbents used today are nitrate[3,4,5], Indigo compounds[1,6,7,8], and 1,2-di(4-pyridyl)ethylene[9,10], and are used as badge samplers or in diffusion tubes. Interest in passive samplers has recently been rekindled by the need to monitor ozone in remote areas and a need to relate exposures to the landscape and forest stand structure. Passive samplers integrate exposure to give a cumulative value. Hourly fluctuations in ozone concentrations that are important to ozone exposure-plant response relationships cannot be determined directly using passive samplers, though efforts are underway to model this using data from the nearest instrumental monitors[11]. In addition, the low cost and flexibility of placement for passive systems make them attractive alternatives for assessing ozone exposure in remote areas and locations that are difficult to access, such as within the forest canopy. Passive samplers may also be used to identify regions with a high frequency of elevated ozone events where additional instrumental monitoring may be required.

Extensive monitoring and characterisation of ozone exposure of forest health plots or spatial extension of forest exposure over the landscape will only be achieved by the use of relatively inexpensive passive samplers. These are being deployed at the sampling sites, with some colocated with available continuous monitors for cross-correlation and calibration purposes[1,12,13]. Employing a large number of passive samplers can provide data on small-scale variations in pollutant exposures**,** a valuable tool in mechanistic studies of air pollution impacts on forest plants[14,15].

Analysis of CanOxy Plate™ samplers (Fig. 1) exposed at plots in forest canopy[1] indicated no relationship with the amounts of ozone (ppmh) monitored at the nearest continuous air quality monitor. These spatial differences, together with our knowledge that strong profiles of ozone exposure were found within the canopy, underlines the importance of *in situ* monitoring of ozone exposure of Forest Health plots[1].

Ozone may also predispose plants to other injuries caused by winter conditions such as thaw-frost cycles[16,17] by reducing root mass and carbon allocation to roots[18]. Monitoring any potential ecological effect of ozone exposure requires the measurement of a number of other air pollutants of concern in order to evaluate the potential for the joint effects of these pollutants[15]. Their possible interaction with climate variables on the health of forest trees may also be important[19]. Here, too, passive samplers may provide a cost-effective way to simultaneously monitor exposure to these other pollutants. Technology has progressed such that we may at least consider these monitoring approaches.

**TABLE 1**
**Examples of Substances Evaluated for their Potential Use as Passive Monitors for Ground-Level Ozone**

| Substance | Reference |
|---|---|
| Alizarin | 24 |
| Alizarin Crimson | 24 |
| Anthraquinone | 24 |
| Curcurmin | 25, 26 |
| 10,10'-Dimethyl-9,9'-biacridylidene | 27 |
| 1,2-Di(4-pyridyl)Ethylene | 9, 10[a] |
| Indigo | 1, 6[a], 25, 26 |
| Indigo carmine | 7[a], 8[a], 23[a], 25, 26 |
| Indigo disulphonate | 28 |
| Various metals | 29[a] |
| 3-Methyl-2-benzothiazoline | 30 |
| Acetone Azine | 31 |
| Nitrate | 3, 4, 32[a] |
| Potassium Iodide | 5, 33 |
| Phenoxazine | 26, 34 |
| Rubber cracking | 29[a], 35 |
| Tin II Diphenylcarbazide | 36, 37 |

[a]Utilized under field conditions

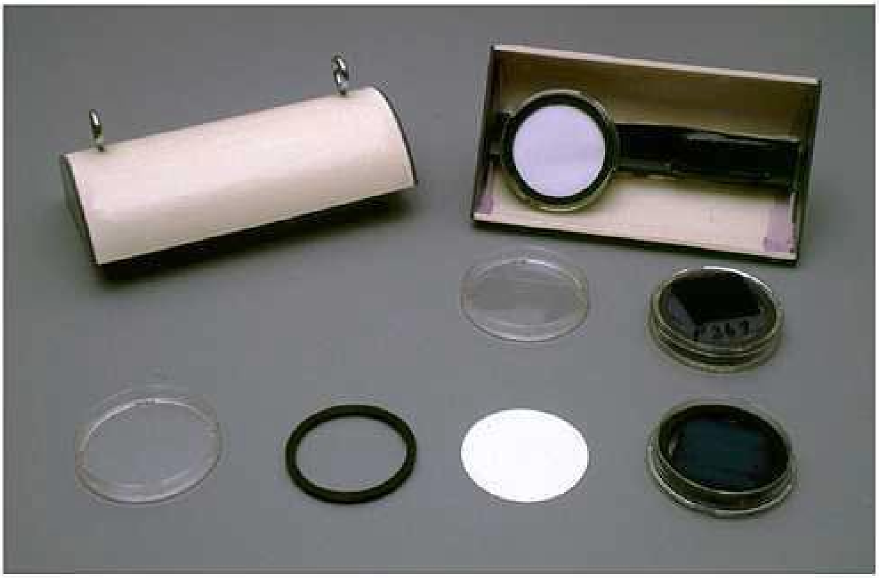

**FIGURE 1.** The CanOxy Plate passive ozone sampler showing the two plates which each have a clear lid and contain a support ring, blue (Indigo) reagent paper, separation ring which separates the paper and the Teflon diffusion membrane, and a retaining ring. The plate is exposed by removing the cap.

Objectives of this report are to demonstrate (1) the efficacy of using the passive ozone sampler for determining temporal and spatial variation in ozone exposure of the forest canopy and (2) the development of a passive ozone sampling protocols and data analysis techniques for forest exposures on different scales.

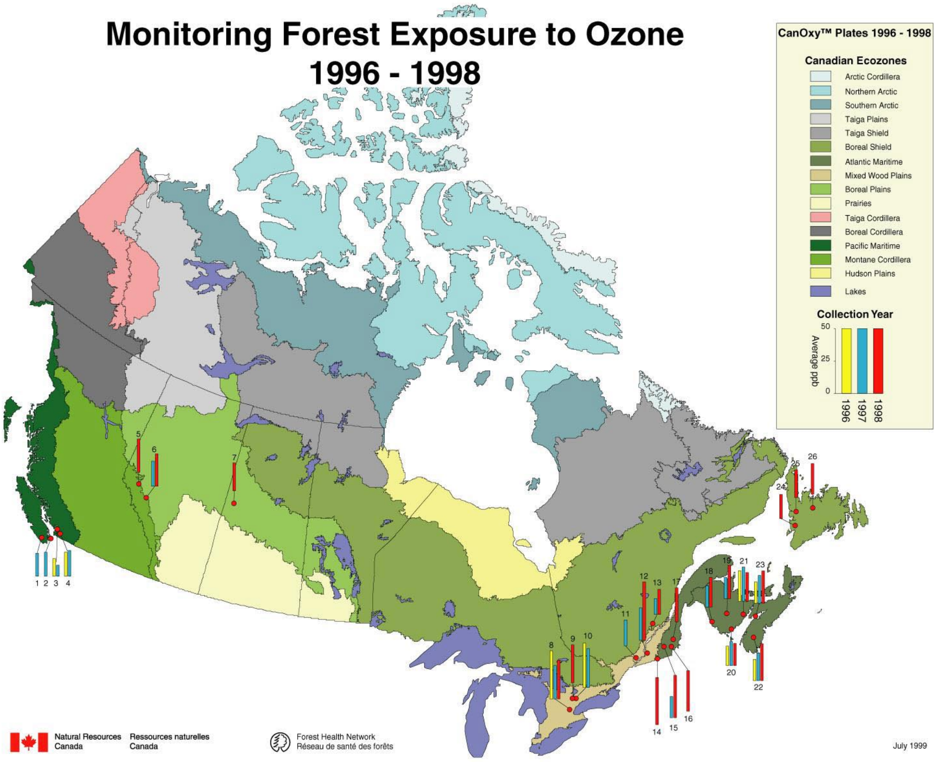

**FIGURE 2.** Locations in the national network of forest health monitoring plots of the Canadian Forest Service–Forest Health Network used in the ozone passive sampling, showing the spatial and temporal variation in canopy ozone exposure over three summers.

## EXPERIMENTAL METHODS/PROCEDURES

## Extensive Ozone Sampling

### *Canadian National Study*

This study was to test the efficacy of using the passive sampler to characterize year-to-year variation and long-term ozone exposure within the forest canopy, with the purpose of relating ozone exposure to forest health parameters. A total of 26 plots were selected (Fig. 2) within a national network of 103 forest health monitoring plots of the Acid Rain National Early Warning System (ARNEWS) established in 1984–1986[20] as part of the Forest Health Network of the Canadian Forest Service. Characterization of summer canopy ozone exposures from 1996–1998 was done for four exposure periods of 2 weeks (mid-July–end of August) using CanOxy Plate™ samplers[1]. Samplers were located one third down from the top of the canopy of representative codominant trees in each plot (Fig. 3) and were also located in the vicinity of continuous ozone monitor sites for the same periods. These co-located arrays of samplers (Fig. 4) were exposed for different time periods during the study, which allowed for the production of field calibrations for quality assurance assessment. Unexposed controls were also placed in sampler shelters for each sampling period to give a reagent blank.

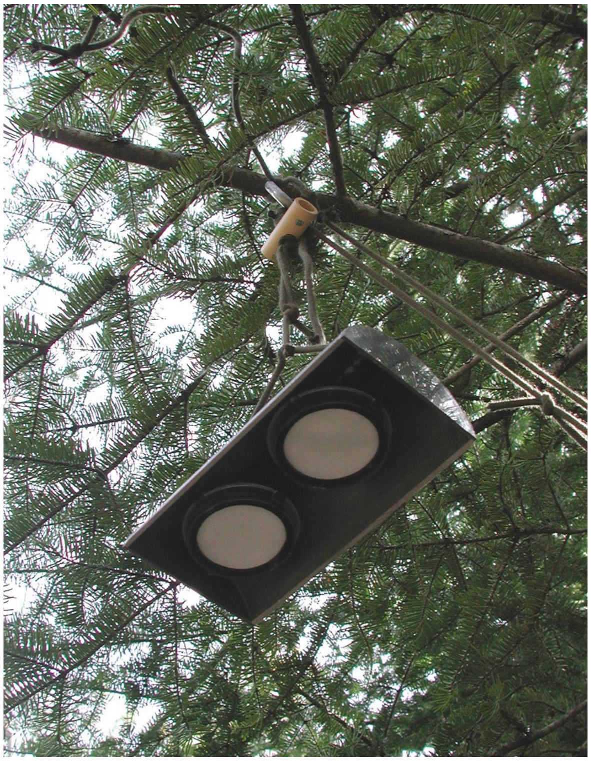

**FIGURE 3.** Installation of a CanOxy Plate passive ozone sampler in the canopy showing the shelter with installed plates, and the pulley system used to retrieve the sampler to exchange the plates.

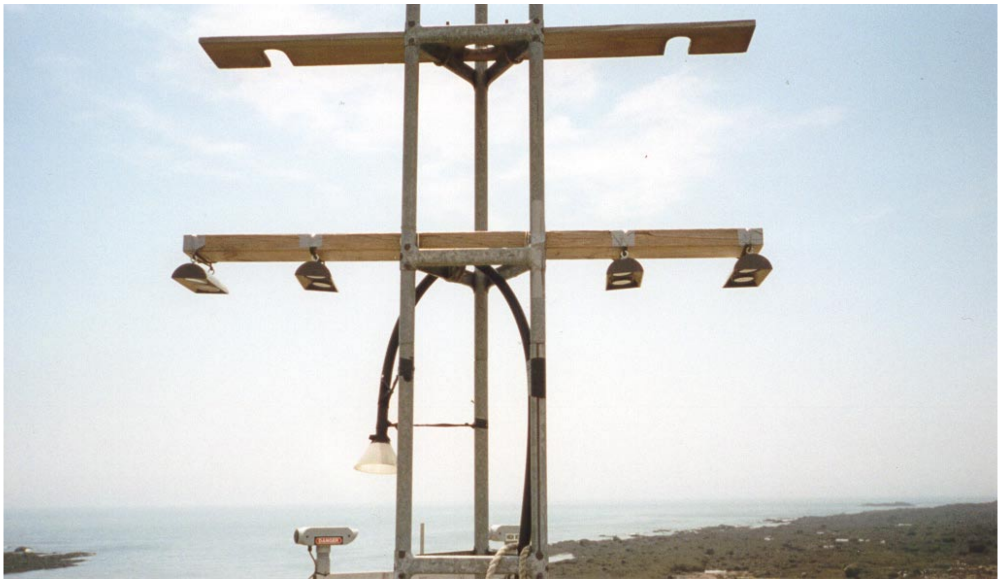

**FIGURE 4.** Installation of the array of CanOxy Plate passive ozone samplers colocated with a New Brunswick provincial air quality monitor (Point Lepreau). These are used to calibrate the canopy samplers.

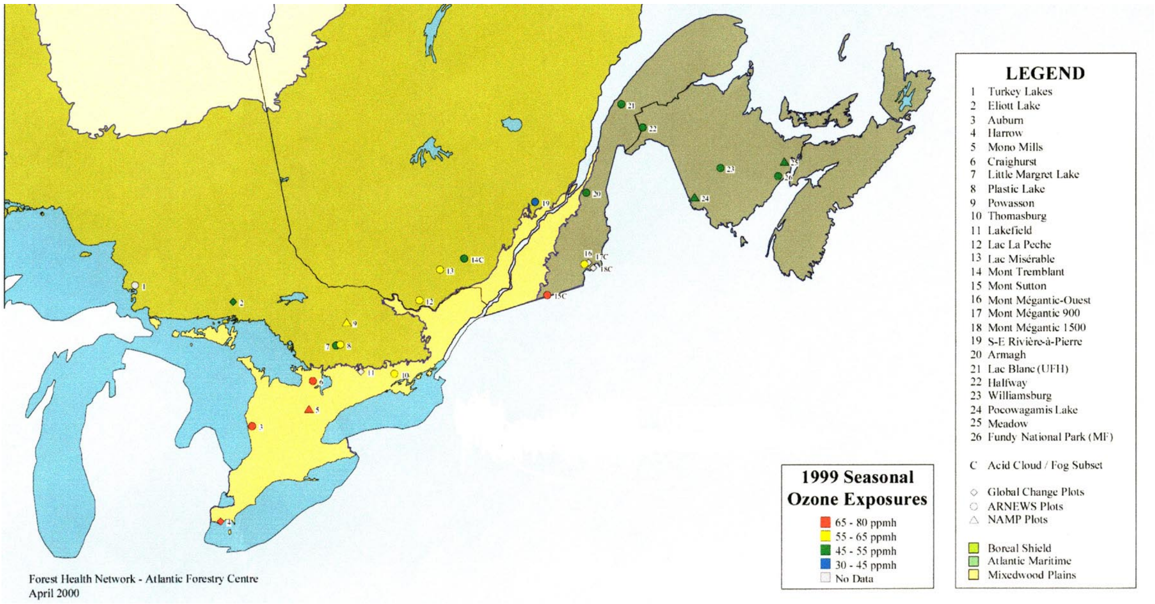

**FIGURE 5.** The locations of the forest plots in the transect used for the Forest Indicators of Global Change (FIGC) project of the Canadian Forest Service–Forest Health Network. This shows the categories for canopy ozone exposure for 1999.

## *Transect Study*

A transect study to determine Forest Indicators of Global Change (FIGC) was established in 1999 using 25 forest health plots in eastern Canada. Fig. 5 classifies the sites based on accumulated ppmh ozone exposures for 1999. These plots followed a known air pollution deposition and climatic gradient that exists from southern New Brunswick to southern Ontario. The deployment of the samplers was similar to that of the ARNEWS study, except that the period of sampling was mid-May to mid-August to include most of the growing season. The mountain sites in Quebec were not accessible until mid-June.

## *Regional Studies*

These types of studies using passive samplers are useful in determining the pattern of ozone distribution and exposure over a province or region where air quality monitoring is often located in urban areas. An early study of canopy exposure in southern New Brunswick was made in 1992 using the CanOxy Plate samplers[12]. Regional patterns in exposure were computed from ozone data from 6 provincial air quality stations and 16 passive sampler stations in the canopy of exposed "edge" trees (ash and black cherry). Weekly mean hourly concentrations were computed as means from samplers placed at the upper and lower canopy, using calibration curves derived from samplers co-located at the nearest suitable air quality monitor. The period of sampler exposure was weekly from late June to mid-August. Contour charts for the weekly mean ozone concentrations were constructed, at that time, using SAS[21] procedures Proc 3DGRID and Proc GCONTOUR, with plot locations entered as cartesian (x,y) coordinates (Figs. 6 and 7).

Another regional scale passive ozone sampling network was established in 1993 with the collaboration with the New Brunswick Department of Environment and Local Government and New Brunswick Power. This network used utility poles as a platform to support the CanOxy Plate$^{TM}$ samplers 10 ft off the ground in 24 locations, mostly in southern New Brunswick (Fig. 8.) The poles were selected on second-class rural roads with minimal traffic. The selected poles were clear of obstructions for at least 25 m and for 50 m in the main upwind vector (SE). The samplers were all exposed for the same 1-week intervals from July 26–August 23 in order to obtain "snap

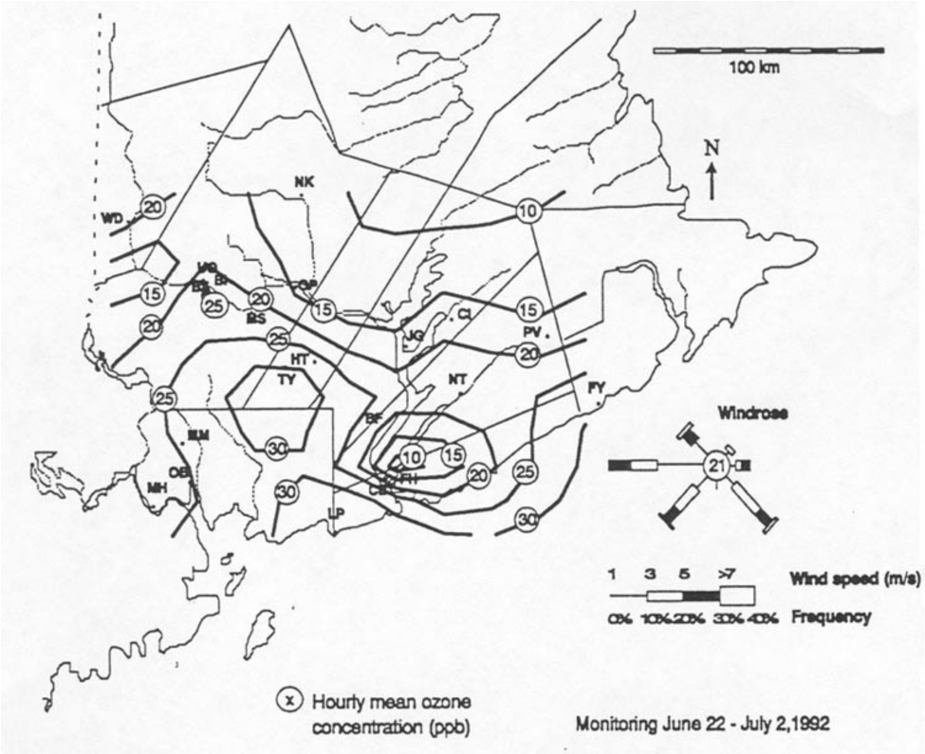

**FIGURE 6.** Contour map of ozone exposure measured with CanOxy Plate passive samplers in the canopy of white ash and black cherry in central and southern New Brunswick with major wind vectors from the southwest.

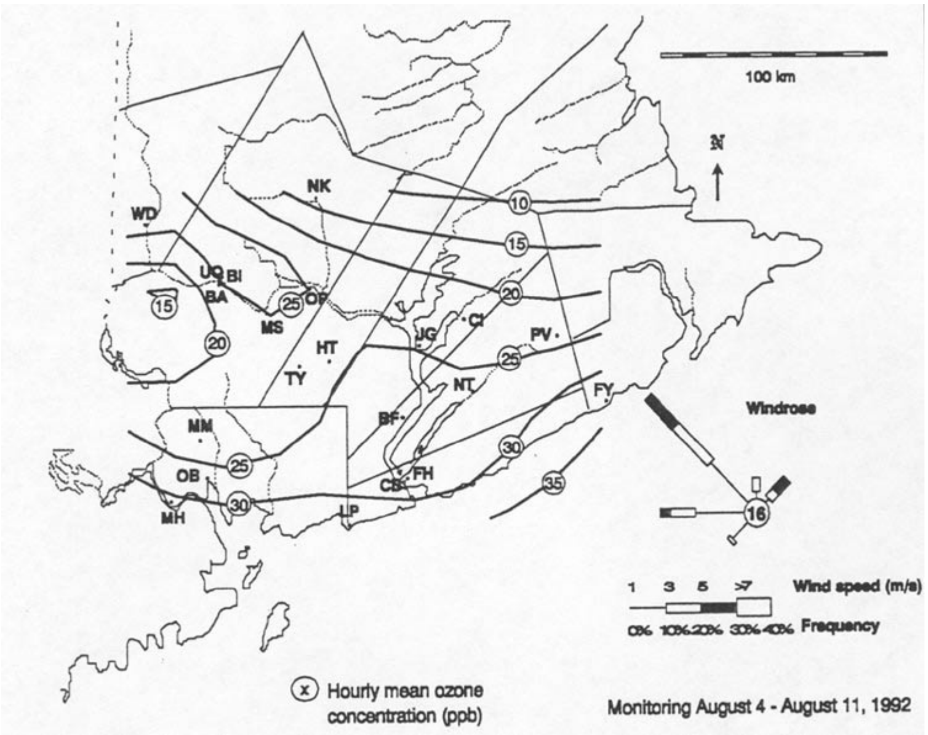

**FIGURE 7.** Contour map of ozone exposure measured with CanOxy Plate passive samplers in the canopy of white ash and black cherry in central and southern New Brunswick with major wind vectors from the northwest.

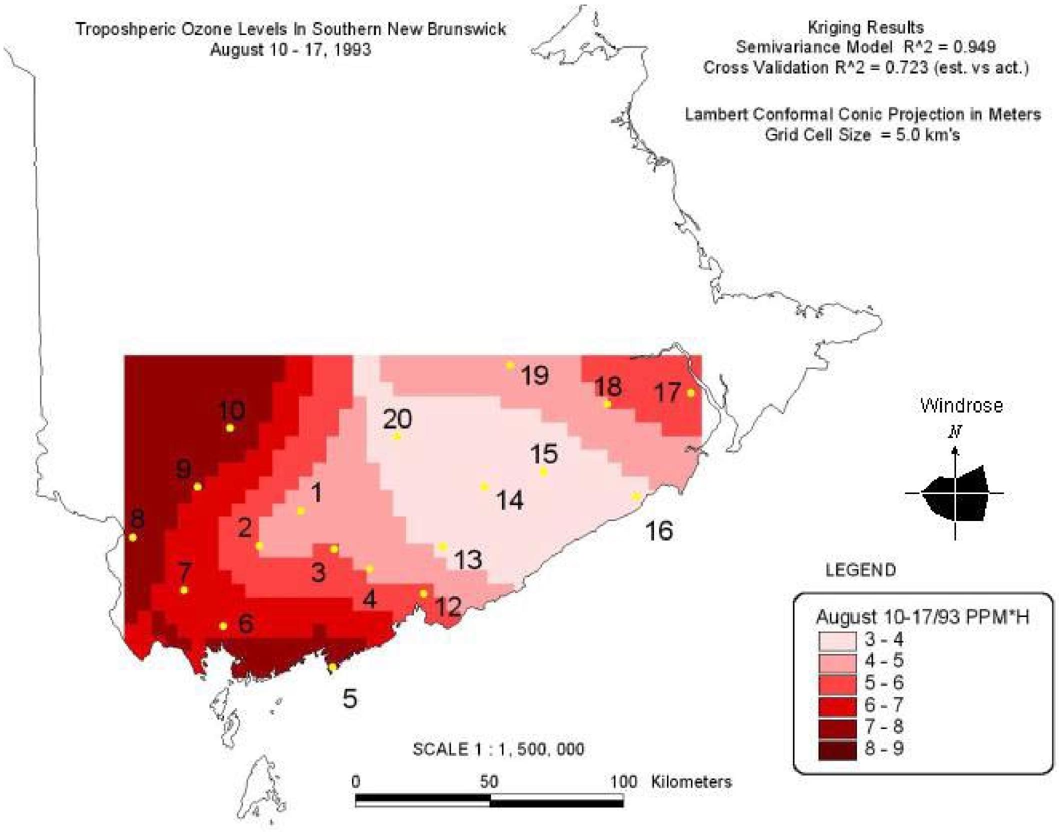

**FIGURE 8.** Kriged surface of ozone exposures measured with CanOxy Plate passive samplers placed on utility poles in southern New Brunswick.

shots" of the ozone climate in rural southern New Brunswick, for determination of potential sites for rural monitoring. The ozone exposures (ppmh) were computed using a calibration line slope derived from samplers colocated at air quality monitors (Blissville, Point Lepreau, and Fundy National Park), and accumulated hourly means from the instrumental monitors over the sampling period. Mean values from the two plates within in the sampler were used.

The sampler sites were georeferenced and registered to a base-map of the province (NRCAN digital database library). The data was formatted and imported into a GIS (Arcview 3.1) projected in meters, then exported for spatial interpolation (GS+) using Kriging techniques. Although the data had minimal suitability regarding geostatistics due to the limited data set (24 sites), some maps with reasonable statistics were produced (Fig. 8).

## *Stand Level Studies*

To understand variation in ozone symptoms and exposure in the canopy, or the exposure of ecosystem components within a forest stand, it is necessary to know the ozone profile within a stand. An investigation of ozone exposure within a mature Mixed Acadian Forest stand was made using a transect through the stand tangentially to the prevailing wind direction[1]. Ozone samplers were placed at known heights in the upper, mid, and lower canopy, and at breast height on selected co-dominant trees, approximately 50 m apart along an overgrown trail from the upwind edge of the stand. Access to the canopy was achieved with the use of a truck fitted with a movable telescopic arm and a bucket.

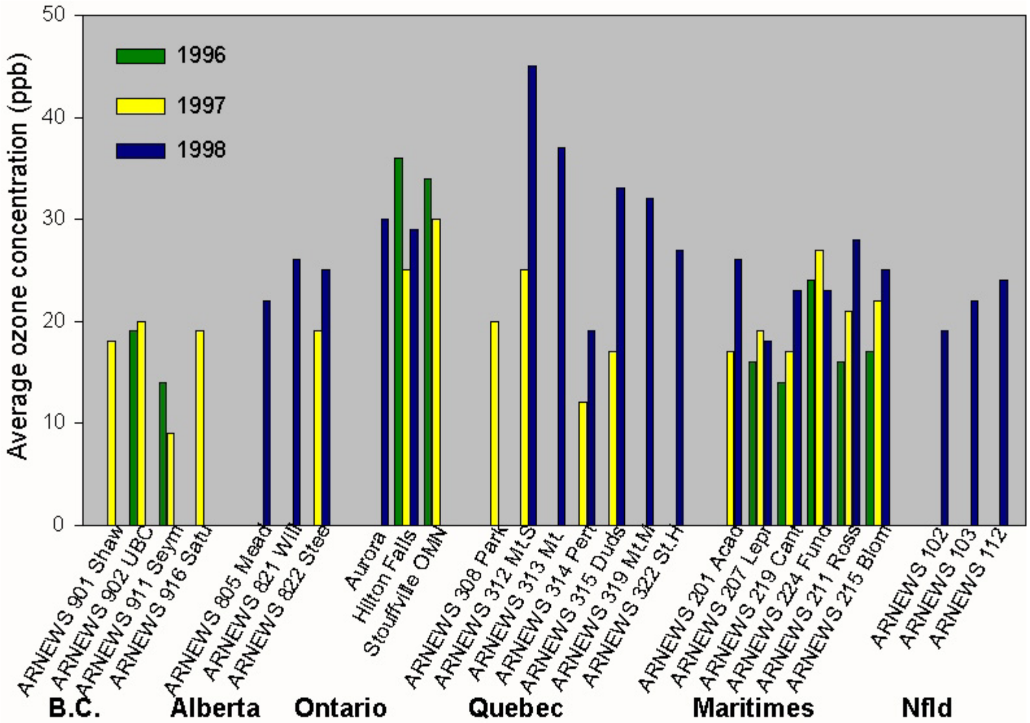

**FIGURE 9.** Ozone exposure values from CanOxy Plate passive samplers placed in the canopy of forest plots in the National Forest Health Monitoring Network of the Canadian Forest Service showing temporal and spatial variation.

## RESULTS AND DISCUSSION

## Extensive Ozone Sampling

The study within the ARNEWS plots showed that there was only a weak relationship between ozone values from samplers in the forest and those at the nearest air quality monitoring site, whereas samplers co-located with the instrumental monitors showed a highly significant relationship ($r = 0.93$)[1]. This indicated the efficacy of monitoring ozone exposure *in situ* within the forest canopy.

The average values sampled over 6 weeks from the forest plot canopies from 1996–1998 (Fig. 9) varied from 9 to 45 ppb, with a mean of 23 ppb. Those compare to the range across all federal and provincial air quality monitors (10 years, 1988–1997) which ranged from 6 to 53 ppb, with a similar mean of 23 ppb, during the same 6-week sampling period (Tom Dann, Environment Canada, personal communication). Using this mean value of 23 ppb as a yardstick, it became clear that 1998 was a high ozone year, with a mean value across all plots of 26.7 ppb. This value may rate along with a known high year 1988, also with a value of 26 ppb, derived as a mean from all instrumental air quality monitors over the same time period. Forest canopies exposed to twice the average concentrations (45 to 46 ppb), i.e., Mt. St. Hilaire, Quebec, and Mt. Sutton, Quebec, with values up to 37 ppb, and Hilton Falls, Ontario, with values up to 36 ppb, would be of special interest as regards forest health effects.

### *Transect Study*

In Fig. 10, 18 sites with a complete record for the growing season are shown (the mountain sites were omitted). These average ozone concentrations ranged from 19 to 37 ppb, indicating that significant variation exists in canopy ozone exposure along the gradient, even when integrated

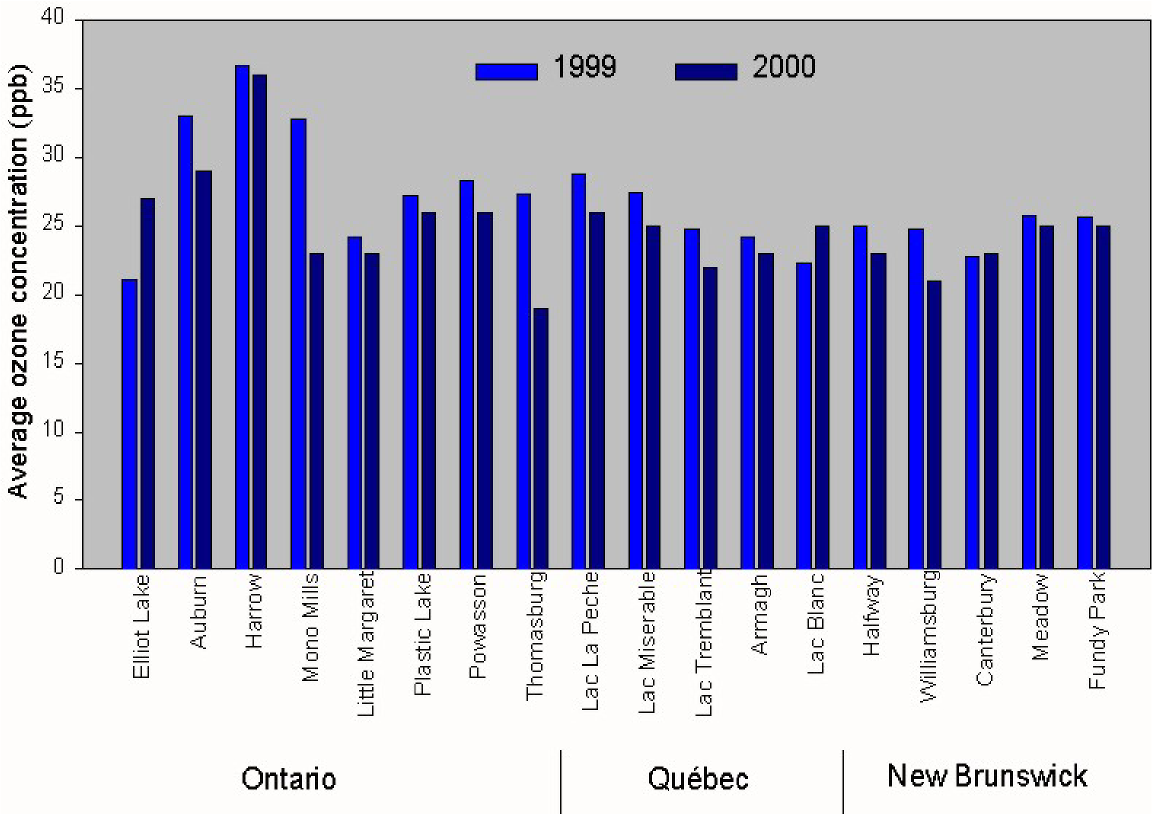

**FIGURE 10.** Ozone exposure values from CanOxy Plate passive samplers placed in the canopy of forest plots in the transect of the Forest Indicators of Global Change (FIGC) project of the Canadian Forest Service–Forest Health Network.

over the growing season and omitting the mountain sites. The highest values are found in southern Ontario at Harrow, Auburn, and Mono Mills, with lower average values in eastern Ontario Quebec and the Maritimes. Year-to-year variation was also evident, especially at the higher ozone exposed sites in Ontario and Quebec. This indicates that, to classify the sites in terms of ozone exposure, sampling should be carried out for a number of years before relating the exposure to long-term forest health effects. Sites, however, with large year-to-year variation may be identified for determination of short-term ozone impacts, such as foliar injury, early leaf senescence, or increased frost damage due to induced changes in frost hardiness.

## Regional Studies

Although a basic type of data interpolation was employed for the spatial analysis at the time (1992), some interesting observations were made using the passive ozone sampler[12]. A distinct gradient to the southeast in decreasing average weekly ozone concentrations was evident until mid-August. This changed to a gradient decreasing to the south as winds moved round from the northwest (e.g., Figs. 6 and 7). An area with lower ozone concentrations was also noted in the vicinity of the city of St. John. Lower values around the city were most likely the result of scavenging of ozone by local sources of NO. It was also observed that as the north-south gradient appeared, the lower concentrations of ozone around the city were no longer evident (Fig. 7). These patterns associated with changes of the major wind vectors of the area (also shown on Figs. 6 and 7) have been related to long-range transport of air pollutants[22]. Simultaneous sampling of $NO_2$ and ozone may help to resolve spatial aspects of their equilibrium reactions in the atmosphere.

The data from the 1993 Southern New Brunswick study had limited suitability as regards geostatistics due to the small number of points[23]. However, some surfaces with reasonable statistics were produced using a grid cell size of 5.0 $km^2$ (i.e., Fig. 8). For this surface, an $r^2$ value of 0.949 was computed for a semivariance model, and the cross validation (estimated vs. actual) $r^2$ was 0.723. This ozone exposure surface was similar to that of the June 1992 study, with (1) a gradient decreasing in ozone to the southeast, (2) a significant area of ozone depletion in the vicinity of St. John, and (3) a strong component wind vectors from the south and east. The area of depletion was a consistent feature in the ozone exposure surfaces produced and is likely due to scavenging by local sources of NO. These regional features in the ozone climate should be taken into consideration when modelling potential effects and exposures in our forests, and demonstrates the efficacy of using passive samplers in dense enough networks to resolve these regional patterns.

## Stand Level Studies

A clear profile in ozone exposure within the Acadian Mixed Wood stand was reported by Cox and Malcolm[1], with a drop of almost 50% over the profile from the top of the canopy to breast height measured with the passive samplers during the 19-day exposure period (Fig. 9). The profile was less developed at the upwind edge of the stand, presumably due to lateral penetration of the air mass. The results indicated that position in the canopy and stand characteristics play an important role in foliage exposure to ozone within the stand. It was concluded that passive samplers have a potentially important role in the empirical determination of foliar exposure and in resolving ozone depletions over the forested landscape.

## ACKNOWLEDGEMENTS

The support of the personnel of the Canadian Forest Service–Forest Health Network across the country was much appreciated. The valuable field assistance of Mr. C. Dickson and the data analysis support of Mr. D. Allen are acknowledged. Financial support through an NSERC student grant (T.P.W.W.) and in-kind support from New Brunswick. Power and the New Brunswick Department of Environment and Local Government were also appreciated.
